# Safety, immunogenicity and preliminary efficacy of multiple-site vaccination with an Epidermal Growth Factor (EGF) based cancer vaccine in advanced non small cell lung cancer (NSCLC) patients

**DOI:** 10.1186/1476-8518-9-7

**Published:** 2011-10-24

**Authors:** Pedro C Rodriguez, Elia Neninger, Beatriz García, Xitlally Popa, Carmen Viada, Patricia Luaces, Gisela González, Agustin Lage, Enrique Montero, Tania Crombet

**Affiliations:** 1 Center of Molecular Immunology. PO Box: 16040, Havana 11600, Cuba; 2 Hermanos Ameijeiras Hospital, Oncology Service, Centro Habana, Cuba

## Abstract

The prognosis of patients with advanced non small cell lung (NSCLC) cancer remains dismal. Epidermal Growth Factor Receptor is over-expressed in many epithelial derived tumors and its role in the development and progression of NSCLC is widely documented. CimaVax-EGF is a therapeutic cancer vaccine composed by human recombinant Epidermal Growth Factor (EGF) conjugated to a carrier protein, P64K from Neisseria Meningitides. The vaccine is intended to induce antibodies against self EGF that would block EGF-EGFR interaction. CimaVax-EGF has been evaluated so far in more than 1000 advanced NSCLC patients, as second line therapy. Two separate studies were compared to assess the impact of high dose vaccination at multiple anatomic sites in terms of immunogenicity, safety and preliminary efficacy in stage IIIb/IV NSCLC patients. In both clinical trials, patients started vaccination 1 month after finishing first line chemotherapy. Vaccination at 4 sites with 2.4 mg of EGF (high dose) was very safe. The most frequent adverse events were grade 1 or 2 injection site reactions, fever, headache and vomiting. Patients had a trend toward higher antibody response. The percent of very good responders significantly augmented and there was a faster decrease of circulating EGF. All vaccinated patients and those classified as good responders immunized with high dose at 4 sites, had a large tendency to improved survival.

## Introduction

In spite of an intensive research effort, lung cancer is the leading cause of cancer death. For advanced non-small-cell lung cancer (NSCLC), first-line platinum-based chemotherapy has reached a plateau of effectiveness [1]. For the second or third line therapy, the reported response rate is usually less than 10% and the median survival time rarely exceeds the 8 months boundary [2]. As a result, searching for new efficacious drugs is warranted.

The Epidermal Growth Factor Receptor is a very well validated target in NSCLC and it is over-expressed in a very high percent of tumors classified as NSCLC [3]. Strategies to block this pathway include tyrosine kinase inhibitors (TKIs) and monoclonal antibodies [2,3]. Erlotinib and gefitinib, 2 small inhibitors, are recommended as second or third line therapies, after the platinum doublet [4]. Moreover, gefitinib has recently been approved in Europe and Japan as frontline treatment of patients bearing EGFR activating mutations [5]. Cetuximab, a chimeric antibody which recognizes the extracellular EGFR domain, can be combined with first line cisplatin/vinorelbine in those subjects with advanced or recurrent NSCLC [6].

Our team is using a different approach to target EGFR consisting on a therapeutic vaccine (CimaVax-EGF) [7]. The vaccine is composed by human recombinant Epidermal Growth Factor (EGF) chemically conjugated to a carrier protein from Neisseria meningitides and emulsified in Montanide ISA51. The vaccine is intended to induce antibodies against EGF, one of the most important ligand of the EGFR, that would block EGF-EGFR binding. So far, 6 clinical trials have been terminated, that proved that the vaccine is safe and able to induce anti-EGF antibodies together with a decrease of EGF concentration in sera [8-14]. However, cancer vaccine optimization is a continuous process devoted to augment the specific immune response. For self antigens, this response should overcome the down-regulation that controls the natural autoimmunity [15]. So far, the strategy to beat the natural tolerance to the EGF has included 4 main directions: the refinement of the adjuvant and carrier [8,9], and the systematic exploration of the schedule and dose dependence [10,13,14].

Previous studies have contributed to delineate CimaVax-EGF components, P64k protein was chosen over Tetanus Toxoid as the carrier molecule [8] and Montanide ISA 51 resulted in a more potent adjuvant as compared to Alum [9,11]. The schedule-dependence of vaccination has been evaluated and several schemes as well as combinations with chemotherapy have been investigated [8-14].

In the randomized Phase II trial, 80 NSCLC subjects received vaccination or best supportive care. Vaccination consists of 0.6 mg of EGF, at 1 injection site. In the efficacy analysis, there was a trend toward survival benefit for all vaccinated patients that became significant in patients younger than 60 years. The survival advantage was also significant in subjects classified as good responders [anti EGF titers ≥ 1: 4000 sera dilution] and in those in whom the EGF concentration declined below 168 pg/ml [13]. Based in the previous evidences from the phase II study and aiming to improve vaccine immunogenicity, a phase III trial was designed with a higher antigen dose, administered at multiple vaccination sites (2 deltoids & gluteus). This Phase III clinical trial is currently ongoing and it is primarily intended to evaluate the efficacy of CimaVax-EGF vs. best supportive care in terms of survival. In this manuscript, we make a comparison of the impact of using high antigen dose distributed in 4 immunization sites (Phase III trial) vs. low dose at 1 injection site (Phase II trial) regarding safety, immunogenicity and preliminary efficacy.

## Materials and methods

### Trial Design

For this analysis, the 40 all vaccinated patients from the phase II clinical trial immunized at a single anatomic site with the EGF vaccine [13] were compared to the first 40 vaccinated patients from a phase III clinical trial, which received vaccination at multiple sites. These 40 patients were evaluated as part of the first interim analysis of the Phase III trial. Patients in both trials signed the informed consent and both protocols were approved by the Institutional Review Boards of the participating institutions.

Both clinical trials, enrolled patients older than 18 years with histology or cytology proven NSCLC at stages IIIB and IV and all patients have had measurable disease at the moment of enrollment. Patients were required to have an Eastern Cooperative Oncology Group (ECOG) performance status (PS) of 2 or less, adequate bone marrow reserve, white blood cells (WBC) count of at least 3, 000/μL, platelet count of at least 100, 000 μL, hemoglobin of at least 10 g/dl, life expectancy of at least 3 months, and creatinine, bilirubin, and transaminase levels according to each institutional standard. Apart from the Phase II trial, in the currently ongoing Phase III study, patients were required to show at least stable disease to first line chemotherapy. On the contrary, 26% of the patients entered Phase II study with a progressive disease, following 4 chemotherapy cycles.

Pregnancy or lactation, secondary malignancies, or history of hypersensitivity to foreign proteins rendered patients ineligible. All patients received 4 to 6 cycles of platinum-based chemotherapy before random assignment and finished first-line chemotherapy regimen at least 4 weeks before entering trial.

### Treatment Schedule

In both trials, a low-dose of cyclophosphamide (200 mg/m^2^), was administered by the intramuscular route, 3 days before the first immunization with CimaVax-EGF (rEGF/rp64k/Montanide ISA 51 VG). An induction phase of 4 quarterly immunizations and monthly re-immunizations was performed. Immunized patients from the phase II clinical trial received vaccination at a single anatomic site, corresponding to 0.6 mg of EGF in 1.2 mL of water in oil emulsion [13]. On the other hand, immunized patients from the ongoing phase III trial, received vaccination at 4 sites (2 deltoids & 2 gluteus), equivalent to 2.4 mg of the antigen, distributed in the 4 anatomic sites, corresponding to 0.6 mg of EGF in 1.2 mL water in oil emulsion per site. Patients assigned to the control arm in both protocols received best supportive care.

### Measurements of Antibody Titers

Blood samples were collected every 14 days for 60 days and monthly thereafter. Anti-EGF antibody titers were measured through an enzyme linked immunosorbent assay (ELISA), as previously described [8]. Anti-EGF antibody titer was defined as the inverse of the highest serum dilution with a final value of optical absorbance equal to two times blank absorbance plus 3 times the SD. Response is provided as the mean of antibodies titers (± S.E.M).

Patients were classified as good antibody responders (GAR) if they reached anti-EGF antibody titers equal or higher than 1:4, 000 sera dilution, and super good antibody responders (SGAR) if patients reached anti-EGF antibody titers at least equivalent to 1:64, 000. An ELISA test was used for the identification of EGF epitopes recognized by sera of immunized patients and EGF serum concentration was measured using a commercial ELISA (Quantikine; R&D Systems Inc, Minneapolis, MN) as previously described [10].

### Statistical Analysis

A geometric T tests for independent samples was used to compare the antibody titers for patients vaccinated under the 2 different schemes. Pearson Chi square was used to compare the demographic categorical variables as well as the percentage of good and super-good responders. Pearson correlation coefficient and Spearman r correlation were used to estimate the correlation between the immunologic. Survival analysis was performed according to the Kaplan-Meier method and the log rank estimate. All analyses were performed using SPSS for windows, version 16.

## Results

Two separate studies were compared to assess the impact of high dose vaccination at multiple anatomic sites in terms of immunogenicity, safety and preliminary efficacy of CimaVax-EGF in advanced NSCLC patients. In both clinical trials, patients started vaccination 1 month after finishing first line chemotherapy. In the Phase II study (40 vaccinated patients), the vaccine dose was 0.6 mg of the antigen, which was administered by the intramuscular route at 1 injection site. The Phase III trial is still ongoing, but for the aim of comparability, we used the data from the first 40 vaccinated patients. These subjects received 4 times the previous dose (2.4 mg of EGF) that was administered by the intramuscular route at 4 anatomic sites.

Before any analysis, patients recruited in the 2 trials were compared in terms of demographic and tumor characteristics. In general, vaccinated patients in both studies were well balanced regarding the most important baseline features (Table 1). All patients had an ECOG PS of 2 or less, stage IIIB was the most represented and the non-adenocarcinoma subtype was the most frequent. Notably, there were more patients younger than 60 years old in the phase II study (low dose/1 injection site) as compared to the Phase III trial (high dose/multiple injection sites).

**Table 1:** Demographic and tumors characteristics of vaccinated patients by study

Demographic Characteristics	Study *(Vaccine Arms)*	
	Phase II Trial	Phase III Trial
**Age**		
Total	40 (100%)	40 (100%)
< 60	30 (75%)	25 (62.5%)
> 60	10 (25%)	15 (37.5%)
**Race**	34 (85%)	32 (80%)
White	34 (85%)	32 (80 %)
African Descendants	1 (2.5%)	6 (15%)
Other	5 (12.5%)	2 (5%)
**Sex**		
F	10 (25%)	27 (31.8%)
M	30 (75%)	57 (68.2%)
Stage		
IIIB	29 (72.5%)	56 (67.4%)
IV	11 (27.5%)	28 (32.6%)
**Histological Type**		
ADC	12 (30.8%)	32 (38.6%)
No ADC	27 (67.5%)	52 (61.4%)
**ECOG**		
0	9 (23%)	35 (40.4%)
1	24 (62%)	43 (50.6%)
2	6 (15%)	6 (7.9%)

### Safety

In both studies, vaccination was very well tolerated. No serious, related adverse events were reported in any of the studies. In the Phase II study [13], the most frequent adverse events consisted on grade 1 or 2 fever, headache, asthenia, chills, tremors, injection site pain and vomiting. On the other hand, the most frequent adverse events in the Phase III were grade 1 or 2 injection site reactions, fever, headache, vomiting, chills and nausea. No significant differences were detected between the 2 vaccination schemes in terms of the frequency or severity of the adverse events.

### Immune response

The humoral anti-EGF response was measured as the principal surrogate marker of the immune response elicited by vaccination. Patients vaccinated with low dose at 1 site at the phase II study reached an anti-EGF antibody titer of 1:3160 sera dilution (geometric mean), while patients from the phase III study reached a anti-EGF antibody titer of 1:7328 (geometric mean; T test p > 0.05).

In addition, in both trials, patients were classified as good antibody responders (GAR) or super good responders (SGAR). GAR and SGAR conditions had been repeatedly correlated with increased survival. Fifty-three percent (52.8%) of the vaccinated patients in the phase II trial were good responders and only 4 patients (10.8%) met the SGAR condition. On the contrary, 56.4% of patients from the vaccine arm in the ongoing phase III study met the GAR criterion while 30.8% were classified as super-responders (SGAR). The percentage of SGAR was significantly higher for patients vaccinated with the high dose, at multiple sites (Table 2).

**Table 2:** Patients' classification according Immune response

Study	Patients' classification		Anti EGF antibody titers *(Geometric mean of sera dilution)*
	GAR	SGAR	
**Phase II Trial** *(low dose, 1 injection site)*	18 (52.8%)	4 (10.8%)	1:3160
**Phase III trial** *(high dose, 4 injection sites)*	22 (56.4%)	12 (30.8%)	1:7328

Patients were classified as Good Antibody Responders (GAR) if they reached an anti-EGF antibody titer ≥ 1:4000 and SGAR if they reached an anti-EGF antibody titer ≥ 1:64000.

**Figure 1:**
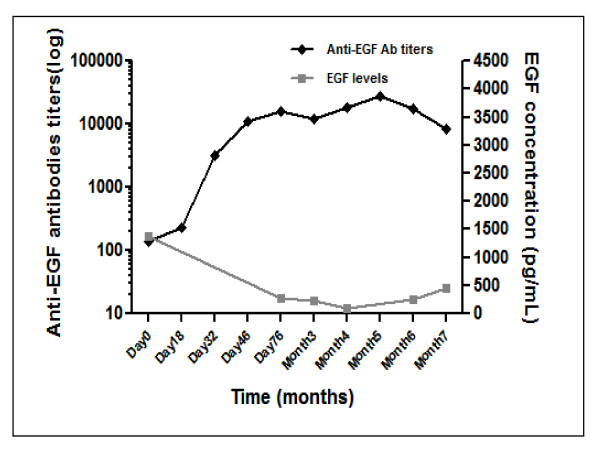
**Kinetic of Anti EGF antibodies and serum EGF concentrations in the Phase III Trial *(Vaccination with high dose/4 anatomic sites)***. The anti-EGF antibody titer was inversely correlated to the EGF serum concentration (spearman r correlation, p < 0.05). in this high dose/multiple sites study the decay below the 500 pg/mL threshold had effect after 76 days of vaccination.

The EGF concentration in serum was also measured as a marker of the vaccine activity. For both immunization schemes, the anti-EGF antibody titer was inversely correlated to the EGF serum concentration (spearman r correlation, p < 0.05). However, the kinetic of the EGF reduction was not the same. In the low dose/1 injection site trial, EGF concentration reduction below a 500 pg/mL took place after vaccinating patients for 10 months, while in the high dose/multiple sites study the decay below the 500 pg/ml threshold had effect after 76 days of vaccination (Figure 1).

As previously reported [14], 46% of the patients showed a predominant response against the B loop of EGF molecule, using the single site vaccination approach. This response was met between months 4 and 7 after starting vaccination. Noticeably, at the same time points, 45.5% of patients in the phase III trial, showed the same immunodominant antibody profile. For both studies, the predominant subclasses were IgG3 and IgG4.

### Preliminary efficacy

The median survival of the vaccinated patients in the phase II trial was 6.47 months and the survival rate at 24 months was 27.27%. The median survival of the first 40 subjects from the phase III trial was 13.57 months and the survival rate at 24 months was 34.2% (log rank p value > 0.05).

Patients who achieved the GAR condition within the Phase II study had a median survival of 11.76 months, while GAR patients in the Phase III survived for 26.87 months (log rank p value > 0.05). Thirty-one percent of patients in the Phase III were classified as SGAR and had a median survival time of 29.9 months, while only 4 subjects in the Phase II achieved this condition, precluding any comparison within the SGAR cohort.

## Discussion

The clinical evaluation of the EGF cancer vaccine started 15 years ago. So far, more than 1000 patients had been immunized worldwide with encouraging results in the treatment of advanced NSCLC and castration resistant prostate cancer patients.

This vaccine is not intended to induce a cellular response but a humoral immunity against EGF, a self protein. The antibodies elicited by vaccination provoke an immune-castration of EGF, which hampers EGF-EGFR interaction. Previous randomized clinical trials had identified the best carrier protein, adjuvant and vaccination schedule. No randomized trials had been conducted so far to assess the impact of immunizing at one vs. 4 anatomic sites with low or high antigen dose. Here we compared 2 separate studies that target the same population (newly diagnosed IIIb/IV NSCLC patients), that started vaccination 1 month after completing first line chemotherapy. The most important distinction between the 2 populations is the response to chemotherapy. All patients had at least disease control in the Phase III, while 26% of subjects in the phase II progressed after first line chemotherapy. Both groups of vaccinated patients were well balanced regarding the remaining important prognostic and predictive factors for the vaccine efficacy. The percent of patients younger than 60 was slightly higher in the Phase II as compared to the Phase III trial. This is precisely the population that had showed the greatest benefit after using CimaVax-EGF. A better result of vaccination in younger people is anticipated, considering the physiologic aging of the immune system, which results in the contraction of the naïve repertoire.

In summary, vaccination at 4 sites with 2.4 mg of EGF was safe and patients had a trend toward higher antibody response and overall survival. The percentage of good responders did not increase and the immune-dominance profile was not modified. However, the percent of very good responders significantly augmented and there was a faster decrease of circulating EGF after vaccinating with higher dose at multiple sites.

Previously, in mice, an EGF vaccine dose resulting suboptimal when administered at a single site, induced a robust immune response if fractionated in 2 or 4 limbs [16]. Administering 4 times the dose at 4 anatomic sites did not increase the immune response of mice.

In our clinical data set, we cannot determine which factor was more relevant for improving vaccine immunogenicity: the amount of antigen or the spatial distribution of the antigen load. The distribution of vaccine inoculation has been previously found to have a significant impact on vaccine potency [17,18]. Theoretically, inoculating a vaccine at multiple sites would increase the total number of precursors that are exposed to the antigen, thereby increasing the number of activated specific effector cells. However, this concept has not been systematically evaluated in the clinical setting.

Conversely, increasing the dose has not always been correlated with greater antigenicity. Many clinical trials have evaluated the impact of dose escalation and there was found to be no direct relationship between dose and immune response [19-21]. Still, reduction of antigen below a minimal threshold can bring the response to a halt and in contrast, persistence of the antigen may stop the immune response through the deletion of effector cells [22]. As a corollary, the optimal dose should be established for each vaccine in the clinical setting.

Even though we have preliminary evidences of improved immunogenicity and clinical benefit of the new vaccination approach, the definitive information will come out after closing enrolment and follow-up of the patients in the ongoing Phase III trial.

Nonetheless, we still have many pending questions: can the immune response be augmented or have we reached a plateau? Could we have the same effect by vaccinating with low dose at multiple sites? Would we induce clonal exhaustion after repeatedly vaccinating with a high dose? Which other manipulations can be done to improve immunogenicity (vaccination in lymphopenia, distinct prime and boosting? So far, a parallel trial evaluating a vaccine-chemotherapy-vaccine schedule is ongoing. The rationale behind is to expand the immune precursors before chemotherapy, to facilitate their preferential homeostatic recovery by re-immunizing after the cytotoxic regimen.

In summary, the evidence of higher immunogenicity and clinical benefit of the new vaccination dose and method is consolidating; the next step would be to confirm if all vaccinated patients had a significantly better survival as compared to controls. The final result of this trial is eagerly awaited.

## Abbreviations used in this paper

ECOG: Eastern Cooperative Oncology Group; EGF: Epidermal Growth Factor; EGFR: Epidermal Growth Factor Receptor; GAR: good antibody-responder; IM: intramuscular; NSCLC: Non- Small Cell Lung Cancer; P64k: P64k carrier protein from Neisseria meningitides; PS: Performance status; sGAR: super-good antibody-responder; WBC: White blood cells.

## Competing interests

The authors declare that they have no competing interests.

## Authors' contributions

PCR coordinated the Phase III trial, designed amendments, completed the trial, processed, analyzed and interpreted data, drafted the manuscript and performed preclinical experiments EN was the principal investigators of both clinical trials, BG and XP carried out the immune assays, CV and PL performed the statistical analysis, GG and AL are the CIMAVax EGF project leaders, EM preclinical experiments project leader, TC participated in the design and coordination of both clinical trials. All authors reviewed and approved the final version of the manuscript prior to its submission for publication.
